# Transcriptomic Differences in Medullary Thyroid Carcinoma According to Grade

**DOI:** 10.1007/s12022-024-09817-0

**Published:** 2024-07-03

**Authors:** Ignacio Ruz-Caracuel, Tamara Caniego-Casas, Teresa Alonso-Gordoa, Irene Carretero-Barrio, Carmen Ariño-Palao, Almudena Santón, Marta Rosas, Héctor Pian, Javier Molina-Cerrillo, Patricia Luengo, José Palacios

**Affiliations:** 1 Pathology Department, Hospital Universitario Ramón y Cajal, IRYCIS, 28034 Madrid, Spain; 2 CIBER-Cáncer (CIBERONC), Madrid, Spain; 3 Medical Oncology Department, Hospital Universitario Ramón y Cajal, IRYCIS, 28034 Madrid, Spain; 4 Medicine School, Alcalá University, 28805 Madrid, Spain; 5 General Surgery Department, Hospital Universitario Ramón y Cajal, IRYCIS, 28034 Madrid, Spain

**Keywords:** Thyroid, Medullary thyroid carcinoma, Neuroendocrine neoplasia, Endocrine pathology, Transcriptomic, DLL3

## Abstract

Medullary thyroid carcinoma (MTC) is a rare cancer derived from neuroendocrine C-cells of the thyroid. In contrast to other neuroendocrine tumors, a histological grading system was lacking until recently. A novel two-tier grading system based on the presence of high proliferation or necrosis is associated with prognosis. Transcriptomic analysis was conducted on 21 MTCs, including 9 high-grade tumors, with known mutational status, using the NanoString Tumor Signaling 360 Panel. This analysis, covering 760 genes, revealed upregulation of the genes *EGLN3*, *EXO1*, *UBE2T*, *UBE2C*, *FOXM1*, *CENPA*, *DLL3*, *CCNA2*, *SOX2*, *KIF23*, and *CDCA5* in high-grade MTCs. Major pathways differentially expressed between high-grade and low-grade MTCs were DNA damage repair, p53 signaling, cell cycle, apoptosis, and Myc signaling. Validation through qRT-PCR in 30 MTCs demonstrated upregulation of *ASCL1*, *DLL3*, and *SOX2* in high-grade MTCs, a gene signature akin to small-cell lung carcinoma, molecular subgroup A. Subsequently, DLL3 expression was validated by immunohistochemistry. MTCs with DLL3 overexpression (defined as ≥ 50% of positive tumor cells) were associated with significantly lower disease-free survival (*p* = 0.041) and overall survival (*p* = 0.01). Moreover, MTCs with desmoplasia had a significantly increased expression of DLL3. Our data supports the idea that DLL3 should be further explored as a predictor of aggressive disease and poor outcomes in MTC.

**Supplementary Information:**

The online version contains supplementary material available at 10.1007/s12022-024-09817-0.

## Introduction

Medullary thyroid carcinoma (MTC) is an uncommon cancer derived from neuroendocrine C-cells of the thyroid. The prognosis is very variable, with an estimated overall 10-year survival of around 70% [1]. Tumor stage is a strong predictor of survival, being the presence of lymph node metastasis as the most relevant prognostic parameter [2].

In contrast to other neuroendocrine neoplasia, a histological classification to grade these tumors was lacking. However, in 2021, a morphological two-tyer grading system was described and proved to have a strong prognostic value. This system classifies tumors as a high grade when any of these features are present: necrosis, ≥ 5 mitosis/2 mm^2^, or a Ki-67 proliferation index ≥ 5% [3]. After its initial description in 2021, this system has been independently validated [4], and it is endorsed by the most recent WHO classification of thyroid neoplasms [5].

The most frequent molecular alterations in MTC are *RET* mutations. These are present in around 50% of sporadic tumors and in virtually all hereditary patients. The second most frequent alterations are *RAS* mutations that constitute approximately 25% of sporadic cases. Thus, around 25% of patients harbor tumors that are negative for *RET* and *RAS* mutations, which are commonly described as “double wild type” [6]. Patients with *RET-*mutated tumors have been described as having a worse prognosis. In addition, recent studies with large cohorts have found an association between *RET* mutation and high-grade MTC [7, 8].

Gene expression studies in MTC have proven useful for characterizing altered molecular pathways in tumors with more aggressive mutations, such as *RET M918T* [9], and for identifying potential therapeutic targets [10]. These studies have also defined molecular subgroups, including a “proliferative-like subtype” characterized by enhanced proliferative activity in juxtaposition to a “mesenchymal-like subtype” characterized by epithelial-to-mesenchymal transition signatures [11]. However, to date, differences in gene expression profiles of MTCs using the new 2021 classification have not been evaluated. Given the prognostic impact of MTC grade, our study explores differences in the expression profile between high- and low-grade MTC.

## Methods

### Patients and Tissue Specimens

The study was approved by the Hospital Universitario Ramón y Cajal Research Ethics Committee (approval code: 85/22). A total of 30 MTC specimens obtained between 2004 and 2022 were included based on tissue availability. Medical records were reviewed for clinical parameters and follow-up. Disease-free survival (DFS) was defined as the time from the date of radical treatment to tumor relapse or death due to any cause, whatever occurred first. Overall survival (OS) was defined as the time from the date of treatment initiation to death due to any cause. Slides were reviewed by two pathologists (I.R.C. and C.A.-P.), and tumor grade was noted according to the International Medullary Thyroid Carcinoma Grading System [3]. Desmoplasia was defined as “newly formed fibrotic (collagenous) stroma surrounding the invasive epithelial tumor cells not found in the non-neoplastic thyroid parenchyma,” as previously described [12]. The presence of amyloid was confirmed with Congo red staining.

### Nucleic Acid Extraction

Nucleic acid extraction was performed after identifying tumor areas containing at least 60% of tumor cells. Macrodissection was performed using formalin-fixed paraffin-embedded (FFPE) blocks. AllPrep DNA/RNA FFPE kit (Qiagen, Valencia, CA, USA) was used to extract DNA and RNA for next-generation sequencing following the manufacturer’s instructions. DNA and RNA were fluorometrically quantified by Qubit dsDNA BR assay kit (Invitrogen, Carlsbad, CA, USA) and Qubit RNA high-sensitivity assay kit (Invitrogen), respectively. Recover All Total Nucleic Acid Isolation Kit (Invitrogen) was used to extract RNA for gene expression analysis following the manufacturer’s instructions. RNA quality was assessed using RNA Screen Tapes on a 2200 TapeStation system (Agilent, Santa Clara, CA, USA).

### Next-Generation Sequencing (NGS)

Mutational analysis was performed with the NGS panel Oncomine Focus Assay (OFA, Thermo Fisher Scientific, Waltham, MA, USA). OFA is a panel that screens 52 genes: it detects hotspot mutations in 35 genes (including *RET, HRAS,* and *NRAS*), copy number variations (CNVs) in 19 genes, and 23 gene fusions (inter- and intragenic). Libraries were automatically prepared using an Ion Chef Instrument (Thermo Fisher Scientific, Waltham, MA, USA). Subsequently, NGS libraries were sequenced with an Ion S5 using the Ion 530 Chef Kit (Thermo Fisher Scientific). Reads were aligned with the GRCh37-hg19 human reference genome. Potential mutations, copy number alteration, and fusions were called using Ion Reporter™ Software (5.10 version-Thermo Fisher Scientific). These processes were performed following the manufacturer’s instructions.

### Gene Expression Analysis

The NanoString nCounter gene expression platform was used to analyze the expression of 760 human mRNAs included in the Tumor Signaling 360 Panel (NanoString Technologies Inc., Seatle, WA, USA) [13]. These mRNAs are involved in the tumor biology, the microenvironment, and the immune response. Each sample requires an input between 100 and 150 ng of total RNA. Fluorescently color-coded reporter probes and biotin-labelled capture probes were hybridized to the mRNA on a thermal cycler overnight and automatically processed and loaded to the NanoString sample cartridge provided in the nCounter Prep Station in accordance with the manufacturer’s protocol.

qRT-PCR was used to validate the expression of selected genes using in-house designed primers (Online Resource 1). After RNA extraction, retrotranscription was performed with the High-Capacity cDNA Reverse Transcription Kit (Thermo Fisher Scientific), following the manufacturer’s instructions. Quantitative PCR was performed using the iTaq Universal SYBR Green Supermix Kit (BIORAD), following the manufacturer’s instructions. Data analysis was performed by quantifying the expression levels of the indicated genes, using a relative quantification ΔΔCt method. The reference gene used was *GAPDH*.

### Immunohistochemistry

Immunohistochemistry (IHC) was performed on whole sections of 4 μm thick mounted on positively charged slides. Antibody against DLL3 (clone SP347, ready-to-use, Ventana, Roche) was incubated for 32 min on a Ventana Benchmark XT Immunostainer (Roche, Basel, Switzerland), after pretreatment with the Cell Conditioning 1 (CC1) solution for 64 min at 100 °C. Antibody against Ki67 (clone MIB-1, ready-to-use, Agilent) was incubated on a Dako Omnis (Agilent).

The Ki67 proliferation index was assessed by two pathologists using a multiheaded microscope. Both pathologists agreed on the hot spot area. Subsequently, one pathologist counted manually between 500 and 2000 tumor cells to determine the ratio of positive cells. DLL3 was quantified independently by two pathologists (I.R.C. and I.C.-B.) blinded to the molecular data of each tumor. Each slide had a small cell lung carcinoma (SCLC) with adjacent non-tumoral lung parenchyma that were used as positive and negative controls, respectively. Moreover, additional slides from selected tumors were incubated without the primary antibody. The percentage of tumor cells was evaluated using the 4 × objective lens, following the methodology previously published [14]. Then, the mean percentage of positive tumor cells of each tumor was obtained, and three groups were considered according to previous reports in MTC [15]: 0%, low (1–49%, and high (≥ 50%).

### Statistical Analysis

NanoString gene expression results were analyzed using nSolver analysis software (NanoString Technologies Inc.). Identification of differentially expressed genes was conducted on normalized data. This software allowed us to obtain the hierarchical grouping, the scatter diagrams (volcano plots), and statistical classification of the differentially expressed genes, along with false discovery rate corrected *p*-values.

Overrepresentation analysis was performed using the “genekitr” package in RStudio v4.2.1 (RStudio, Boston, MA, USA) on the differentially expressed genes. These genes were queried in the curated pathways from the Reactome database contained within the Molecular Signatures Database (accession date: June 5, 2024). Numerical variables were summarized as median (range) and categorical variables as frequencies and percentages. The chi-squared or Fisher’s exact test was used to evaluate the association between qualitative variables. Mann–Whitney’s *U* test was used to evaluate the association between quantitative variables. DFS and OS data were plotted in Kaplan–Meier curves, and the log-rank test was used to compare these parameters. Data were analyzed using the statistical software IBM SPSS v19 (IBM, Armonk, NY, USA) and RStudio was employed for graphical representation. The threshold for statistical significance was set at *p* < 0.05.

## Results

### Cohort Characteristics

A total of 30 patients with MTCs were included in the current study. There were 11 (36.7%) patients with high-grade MTCs and 19 (63.3%) with low-grade MTCs. Table 1 shows the clinicopathological characteristics of the cohort according to the MTC grade. The median age was 50.5 years (range: 24–84 years) with a female-male ratio of 2:1. Ten (90.9%) patients with high-grade MTCs had lymph node metastases at diagnosis compared to 6 (31.6%) patients with low-grade MTCs (*p* = 0.002). In contrast, no patients in the high-grade subgroup were diagnosed at AJCC stage I compared to 8 (42.1%) patients with low-grade MTCs.

**Table 1:** Clinicopathological features of patients with MTC

Parameter	All (*n* = 30)	High grade (*n* = 11)	Low grade (*n* = 19)	*P* value
Clinical features				
Age, years, median (range)	60.5 (24–84)	56 (24–77)	62 (33–84)	0.420
Female to male (ratio)	20:10 (2:1)	4:7 (0.57:1)	16:3 (5.33:1)	0.150
Lymph node metastases at diagnosis (%)	16 (53.3%)	10 (90.9%)	6 (31.6%)	0.002
AJCC stage (%)				0.012
I	8 (26.7%)	0	8 (42.1%)	
II	3 (10.0%)	1 (9.1%)	2 (10.5%)	
III	3 (10.0%)	0	3 (15.8%)	
IV	16 (53.3%)	10 (90.9%)	6 (31.6%)	
Relapses (%)	7 (25.9%)	6 (75.0%)	1 (5.3%)	0.001
Pathological features				
Tumor size, cm, median (range)	2 (0.5–11.5)	5 (1.2–11.5)	1.5 (0.5–4.2)	< 0.01
Vascular invasion (%)	11 (36.7%)	10 (90.9%)	1 (5.3%)	< 0.001
Microscopic extrathyroidal extension (%)	10 (33.3%)	8 (72.7%)	2 (10.5%)	0.001
Macroscopic extrathyroidal extension (%)	8 (26.7%)	8 (72.7%)	0	< 0.001
Positive resection margin (%)	6 (20.0%)	6 (54.5%)	0	0.001
Desmoplasia (%)	19 (63.3%)	10 (90.9%)	9 (47.4%)	0.023
Tumor necrosis (%)	8 (26.7%)	8 (72.7%)	0	
Mitotic index ≥ 5 per 2mm2 (%)	3 (10.0%)	3 (27.3%)	0	
Ki67 proliferation index ≥ 5% (%)	8 (26.7%)	8 (72.7%)	0	
Somatic mutations				
*RET*	16 (53.3%)	9 (81.8%)	7 (36.8%)	
*RET M918T*	12 (40.0%)	9 (81.8%)	3 (15.8%)	
*RAS*	5 (16.6%)	2 (18.2%)	3 (15.8%)	
*RET* and *RAS* wildtype	3 (10.0%)	0	3 (15.8%)	
Unknown	6 (20.0%)	0	6 (31.6%)	

Regarding pathological features, high-grade MTCs were larger than low-grade MTCs (median: 5 cm vs 2 cm, respectively, *p* value < 0.01). High-grade MTCs showed a higher proportion of tumors with vascular invasion (*p* < 0.001), microscopic extrathyroidal extension (*p* = 0.001), macroscopic extrathyroidal extension (*p* < 0.001), positive resection margin (*p* = 0.001), and desmoplasia (*p* = 0.023). Necrosis represented less than 1% of the tumor area, except in one tumor where it accounted for up to 30% of the area.

Mutational status was analyzed in 24 MTCs including all high-grade MTCs. Cases without this information included five tumors measuring less than 1 cm and one tumor that did not reach the quality parameters. The distribution of mutations was as follows: 16 *RET* mutations (12 *RET* p.M918T, 1 *RET* p.C630I, 1 *RET* p.C634Y, 1 *RET* p.A883F, and 1 *RET* p.D898_E901del), 3 *HRAS* p.Q61R mutations, and 2 *KRAS* mutations (1 *KRAS* p.Q61R and 1 *KRAS* p.G12V). Three tumors with *RET* p.M918T mutations harbored additional mutations. One high-grade MTC had a *PIK3CA* p.E39K. Another high grade had a *PIK3CA* p.D1045N and a *BRAF* p.T599I. A low-grade MTC had a *JAK1* p.V658I and a *DDR2* p.R124W (Online Resource 2). Germline *RET* status was not known.

### Differential Gene Expression Between High-Grade and Low-Grade MTC

**Fig. 1:**
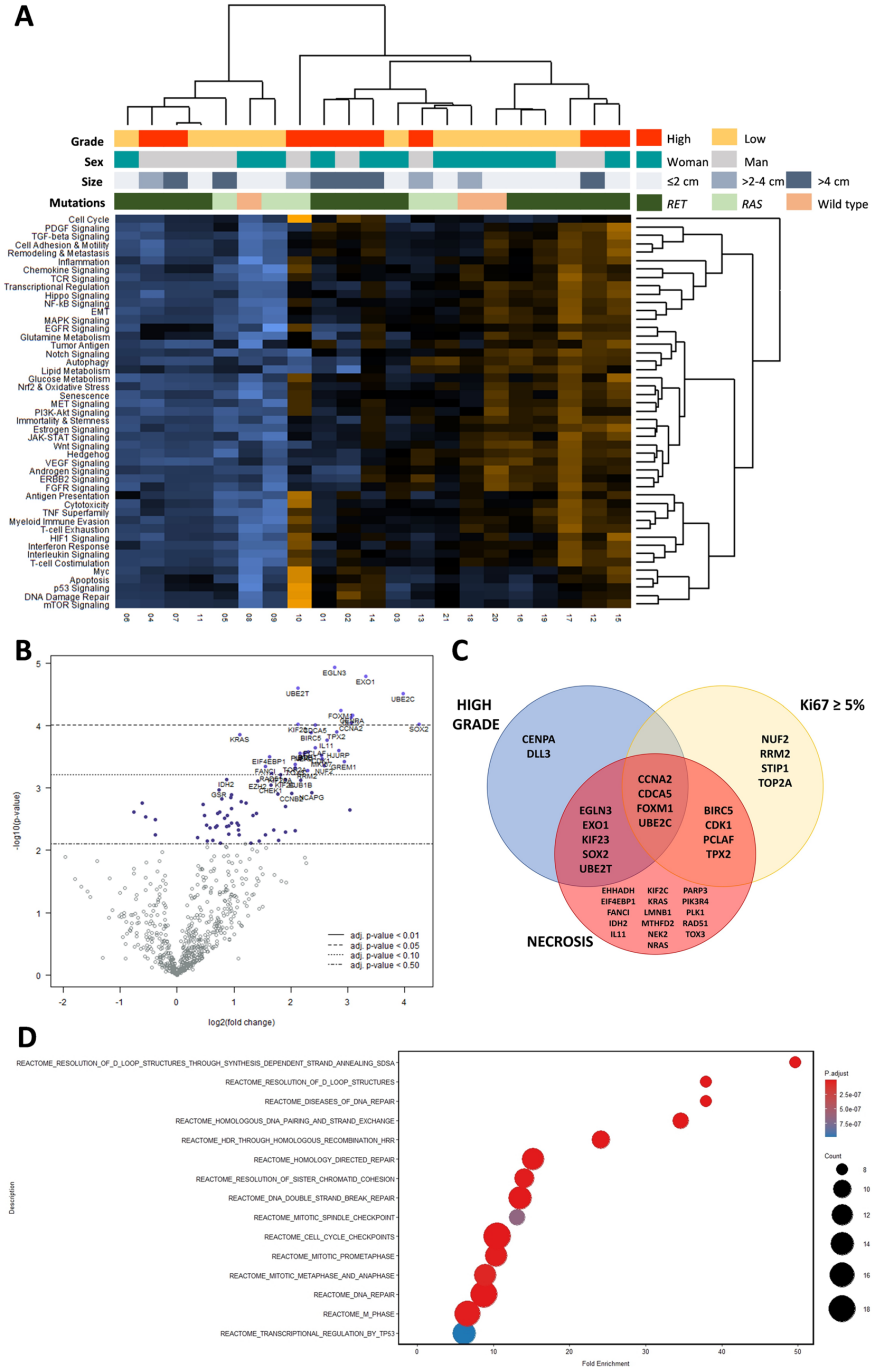
Expression profile according to MTC grade. **A** Unsupervised clustering analysis based on molecular pathways did not cluster separately high-grade from low-grade MTC. **B** Volcano plot showing differentially expressed genes according to grade. **C** Venn diagram including the genes differently expressed among three morphological conditions: high-grade vs low-grade MTC, proliferation index ≥ 5% vs < 5%, and tumors with necrosis present vs absent. **D** Top fifteen overrepresented pathways considering the genes differentially expressed between high-grade and low-grade MTC

Transcriptomic analysis using the NanoString platform was performed in 21 MTCs with available mutational status. Representative tumor areas from 9 high-grade and 12 low-grade MTCs were included. Unsupervised clustering analysis was not able to segregate high-grade from low-grade MTCs (Fig. 1A). There were eleven differentially expressed genes according to grade: *EGLN3*, *EXO1*, *UBE2T*, *UBE2C*, *FOXM1*, *CENPA*, *DLL3*, *CCNA2*, *SOX2*, *KIF23*, and *CDCA5* (Fig. 1B). Moreover, we analyzed transcriptomic differences according to the two morphological elements that give rise to the grade: the presence of necrosis and a high proliferation index defined by a Ki67 ≥ 5% (Online Resource 3). The Venn diagram showed that there were only two genes exclusively overexpressed in the high-grade MTC subset: *DLL3*, an inhibitory NOTCH pathway ligand associated with neuroendocrine carcinomas [16], and *CENPA*, a protein-coding gene associated with centromere function (Fig. 1C). In contrast, there were four genes (*CCNA2*, *CDCA5*, *FOXM1*, and *UBE2C*) differentially expressed among those three conditions.

The most significant pathways differentially expressed between high-grade and low-grade MTCs were DNA damage repair (*p* = 0.013) and p53 signaling (*p* = 0.013); followed by cell cycle (*p* = 0.016), apoptosis (*p* = 0.023), Myc signaling (*p* = 0.028), and mTOR signaling (*p* = 0.047). In addition, we conducted an independent overrepresentation analysis considering the eleven differentially expressed genes according to grade. As shown in Fig. 1D, the fifteen most overrepresented pathways from the Reactome database were related to cell cycle progression and DNA repair.

**Fig. 2:**
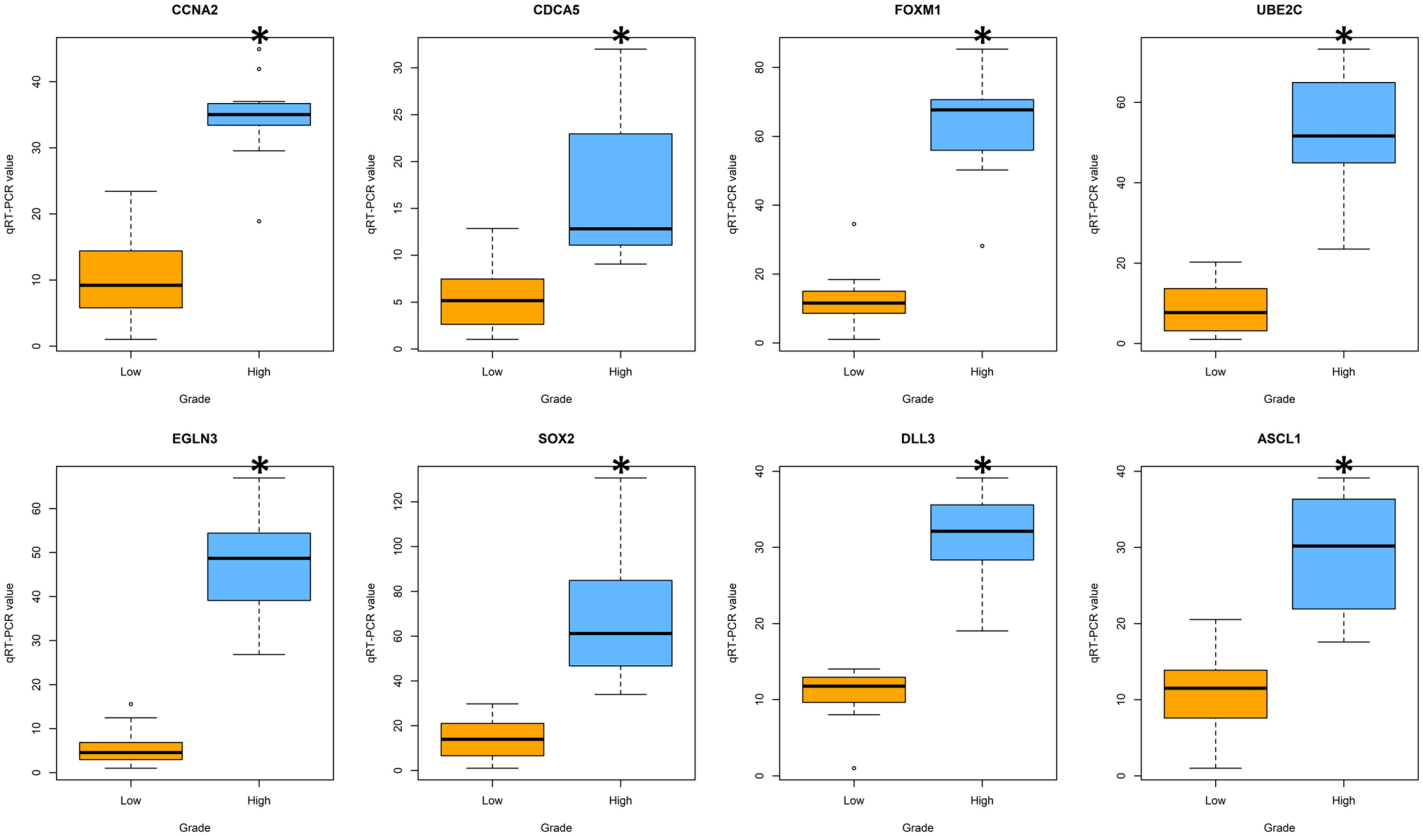
Panel of genes validated by qRT-PCR. *p < 0.001

A panel of eight genes was further validated by qRT-PCR in 25 MTCs (11 high grade and 14 low grade) (Fig. 2). This panel included the four genes overexpressed in the three conditions (*CCNA2*, *CDCA5*, *FOXM1*, and *UBE2C*); *EGLN3*, the most overexpressed gene in high-grade MTC; *SOX2*, a cancer stem cells related transcription factor overexpressed in high-grade follicular-derived thyroid carcinomas [17]; the two genes overexpressed exclusively in a high-grade subset (*CENPA* and *DLL3*); and *ASCL1*, not included in the NanoString Tumor Signaling 360 Panel. *ASCL1* is a known upstream regulator of *DLL3* expression [18]. qRT-PCR validation showed the eight genes to be overexpressed in high-grade MTC compared to low-grade MTC (*p* < 0.001) (Fig. 2). Moreover, there was a positive correlation between the levels of *DLL3* and *ASCL1* (*r* = 0.77, 95% C.I. 0.54–0.89, *p* = 6.738e-06), and between the levels of *SOX2* and *ASCL1* (*r* = 0.73, 95% C.I. 0.46–0.87, *p* = 3.963e-05) (Online Resource 4).

### DLL3 Expression Impact on Prognosis

**Fig. 3:**
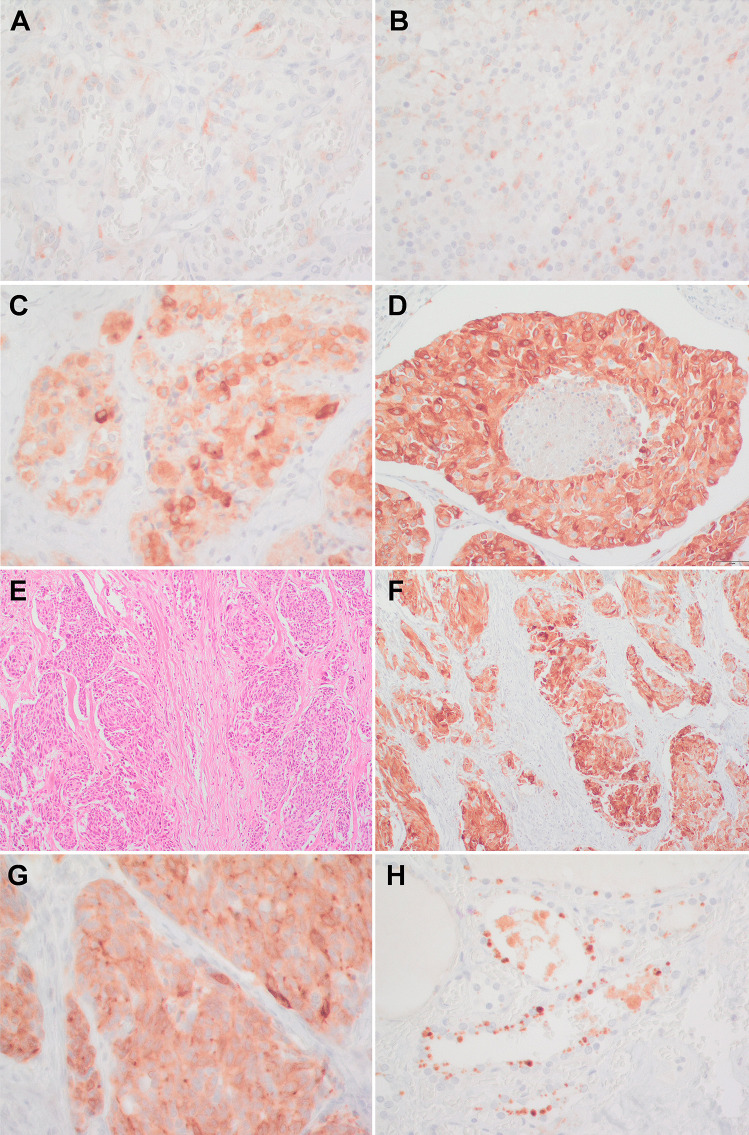
DLL3 immunohistochemical expression in MTC. **A** Low-grade MTC and **B** high-grade MTC with low expression of DLL3 (DLL3, 40 ×). **C** Low-grade MTC with a high cytoplasmic expression of DLL3 of variable intensity among tumor cells (DLL3, 40 ×). **D** High-grade MTC with high expression of DLL3 and central comedonecrosis (DLL3, 20 ×). **E** High-grade MTC with prominent desmoplasia (H-E, 10 ×) and **F** a high expression of DLL3 (DLL3, 10 ×). **G** Strong perinuclear dot-like staining in scattered cells (DLL3, 40 ×). **H** Non-specific granular staining in normal follicles (DLL3, 40 ×)

DLL3 is a protein implicated in neuroendocrine tumorigenesis and is known to be upregulated in SCLC subtype A (A means associated with *ASCL1* upregulation) [19]. Since this protein is being explored as a target for conjugated drugs, DLL3 expression by IHC has been extensively studied in SCLC [14]. Therefore, we further quantified DLL3 protein expression by IHC. DLL3 diffusely stains the cytoplasm of tumor cells in MTC with variable intensity (Fig. 3A–F). Interestingly, a perinuclear strong dot-like staining was observed in scattered cells in some tumors (Fig. 3G). The non-tumor thyroid was negative for DLL3 expression, except for granular staining related to colloid content observed in some normal follicles (Fig. 3H). This granular staining was interpreted as an artifact because it was also present, though with weaker intensity, in slides incubated without the primary antibody. The proportion of positive tumor cells was measured, as previously described for SCLC [14]. There was a tendency towards an increased number of high-grade tumors with high expression of DLL3, although it was non-significant (Table 2). The presence of desmoplasia was associated with higher expression of DLL3 (Fig. 3). In tumors with desmoplasia, the mean expression of DLL3 was 55.18 ± 32.03, in contrast to a mean expression of 14.36 ± 21.58 in tumors without desmoplasia (*p* = 0.01). Nevertheless, considering the 24 tumors with a known mutational status, there were no significant differences in DLL3 expression between *RET* mutated tumors (50.09 ± 35.85) compared with *RET* wild type (28.88 ± 30.89) (*p* = 0.157).

**Table 2:** DLL3 expression according to MTC grading

Percentage DLL3 expression	Low grade (%)	High grade (%)	All (%)	*P* value
Null (< 1%)	4 (21.1%)	1 (9.1%)	5 (16.7%)	0.228
Low (1–49%)	9 (47.4%)	3 (27.3%)	12 (40.0%)	
High (≥ 50%)	6 (31.6%)	7 (63.6%)	13 (43.3%)	

**Fig. 4:**
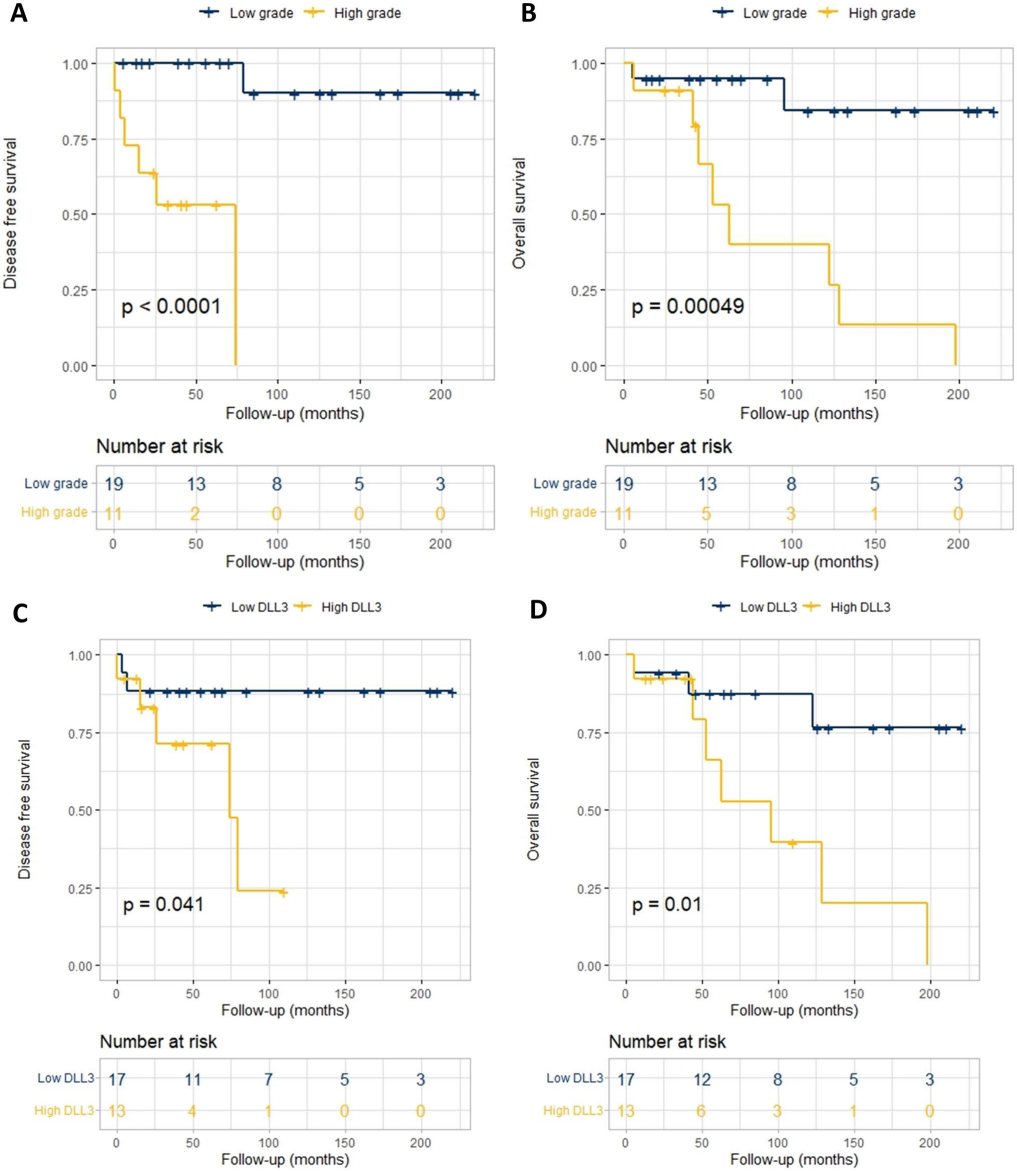
Kaplan–Meier survival curves according to grade and DLL3 expression. **A** Disease-free survival according to grade. **B** Overall survival according to grade. **C** Disease-free survival according to DLL3 positive expression. **D** Overall survival according to DLL3 positive expression

Because we had survival data from our patients, we calculated DFS and OS by taking the cutoff of ≥ 50% positive tumor cells as high expression of DLL3, as previously considered in MTC [14, 15]). MTC grade had an impact on prognosis. After a median follow-up of 63.87 months (range 5.57–220.53 months), high-grade MTC showed a significantly worse prognosis in both, disease-free survival (*p* < 0.0001) and overall survival (*p* < 0.00049), compared with low-grade MTC (Fig. 4A, B). DLL3 overexpression, defined as ≥ 50% of positive tumor cells, was also associated with a significantly lower disease-free survival (*p* = 0.041) (Fig. 4C) and overall survival (*p* = 0.01) (Fig. 4D). The hazard ratio was not calculated because there were few or no events in the groups with the most favorable prognosis.

## Discussion

The MTC grading system is a novel prognostic parameter that identifies patients at a higher risk of relapse and lower overall survival. Going deeper, we have additionally shown that there are transcriptomic differences between high-grade and low-grade MTC.

Using the NanoString Tumor Signaling 360 Panel, we were able to identify eleven genes differentially expressed between high-grade and low-grade MTC. The grade is based on proliferation activity and necrosis. This is consistent with the finding that some of the most differentially expressed pathways are DNA damage repair, cell cycle, and apoptosis. Other pathways overexpressed in high-grade MTC were p53 signaling and Myc signaling. It is interesting that Qu et al. [11] using RNA-seq from MTCs identified a proliferative subtype that upregulated Myc targets and DNA repair genes. This cluster, identified by unsupervised clustering analysis, comprised most patients with relapse in their series, and it partly agrees with those pathways identified by us in high-grade MTC. Therefore, the Myc signaling pathway seems to play a role in aggressive MTC as occurs in SCLC [20]. In addition, the p53 pathway has been described as upregulated in MTCs harboring *RET* M918T mutation, considered to be an aggressive subgroup [9]. Furthermore, *TP53* mutations have recently been identified as an adverse molecular event associated with decreased overall survival in MTC [7]. The fact that we have demonstrated for the first time an association of the upregulation of some genes such as *EGLN3*, *SOX2*, and *DLL3* with aggressive MTC was due to our novel approach, considering morphological grade to stratify patients instead of *RET* mutational status [9, 10], as well as from using a panel of genes focused on cancer pathways instead of RNA-seq. Interestingly, all our high-grade *RET*-mutated MTCs harbored the *RET* M918T mutation, which was also present in 3 low-grade MTCs.

*EGLN3* was the most differentially expressed gene between both conditions. *EGLN3* encodes for a prolyl hydroxylase, also known as PHD3. It is involved in a variety of processes such as HIF factors regulation [21], apoptosis induction [22], and p53 protein stabilization [23]. Its prognostic value in cancer is not universal. For example, in clear cell renal carcinoma, where HIF induction has a pathogenic role, *EGLN3* is overexpressed. However, its overexpression has been associated with a better recurrence-free survival [24]. In contrast, there are conflicting reports on non-small-cell lung cancer regarding its prognostic value [25, 26]. In our series, *EGLN3* overexpression was associated with the presence of necrosis but not with a high proliferation index. Morphological necrosis in MTC is characterized by the presence of debris and apoptotic cell figures, so *EGLN3* overexpression could reflect increased apoptosis. *EGLN3* is induced by hypoxia and suppressed HIF activity by hydroxylating HIF1α and HIF2α to target them to proteasomal degradation [27]. However, in our panel, *HIF1α* was not associated with grade, nor with the presence of necrosis. In this regard, HIF1α expression has been previously associated with a lower overall survival and progression-free survival in MTC [28]. This is the first report of *EGLN3* associated with a worse prognostic parameter in MTC.

The four genes that were overexpressed in the three morphological conditions (high grade, high proliferation, and the presence of necrosis) are genes required for cell cycle progression: *Cyclin A2 (CCNA2), Cell Division Cycle Associated 5 (CDCA5), Forkhead Box M1 (FOXM1)*, and *Ubiquitin Conjugating Enzyme 2C (UBE2C).* The upregulation of these genes is expected in conditions with increased proliferation, and they are commonly identified as worse prognostic parameters in many cancer datasets [29, 30]. For example, *UBE2C* participates in the regulation of cell cycle progression through the M phase. Its overexpression has been associated with a release from the inhibitory signal of mitotic spindle checkpoint and reentry into mitosis. Moreover, it can induce an uncontrolled anaphase-promoting complex/cyclosome (APC/C) activity that led to chromosome missegregation and progressive aneuploidy [31]. *UBE2C* upregulation has been found in cancers from many origins, including follicular cell–derived thyroid carcinomas [31, 32].

The gene *sex-determining region Y-box 2 (SOX2)* was also upregulated in high-grade MTC and MTC with necrosis. This gene has a main role in the maintenance of the stem cell phenotype and self-renewal, being one of the Yamanaka factors [33]. In addition, it is considered an oncoprotein that controls several features including uncontrolled proliferation, resistance to apoptosis, cancer stem cell formation, migration, invasion, and epithelial-to-mesenchymal transition. In most cancer types, it is associated with poor prognosis and high tumor grade [34, 35], and it is expressed in anaplastic thyroid carcinoma, a follicular-derived thyroid neoplasia with an ominous prognosis [36]. To our knowledge, this is the first report of an association between *SOX2* overexpression and high-grade or aggressive features in MTC.

Two genes were upregulated in high-grade MTC that did not appear in the subsets of proliferation or necrosis: *CENPA* and *DLL3*. *CENPA* encodes for a histone H3-like nucleosomal protein found in centromeric nucleosomes, which is also required for the assembly of kinetochore proteins, so it is implicated in cell cycle progression [37, 38].

*DLL3* encodes for Delta-like 3 protein (DLL3), an inhibitory ligand of the Notch signaling pathway. Neuroendocrine carcinomas, such as SCLC, express DLL3 in more than 85% of tumors [14], and in the digestive system, DLL3 is overexpressed in neuroendocrine carcinomas compared with neuroendocrine tumors [39]. *DLL3* is a recognized downstream target of *achaete-scute homologue 1 (ASCL1)*, which is a key factor in neuroendocrine cell differentiation. Moreover, *ASCL1* upregulation defined the most frequent molecular subtype of SCLC (SCLC-A), which also upregulated *DLL3* and *SOX2* [19, 40]. In our series, both *DLL3* and *SOX2* correlate with *ASCL1* expression, being the three genes upregulated in high-grade MTC.

Taking into consideration the MTC mutational status, there was no association between the presence of *RET* mutation and the level of expression of DLL3 by IHC, although there was a trend towards overexpression in *RET*-mutated tumors. This is an issue that should be examined further in a larger cohort of patients.

The presence of desmoplasia in MTC has been associated with the presence of lymph node metastases, and it has been proposed as a parameter to report to reduce lymph node dissections in patients with low risk of lymph node metastases [12, 41, 42]. Moreover, DLL3 expression has already been associated with the presence of desmoplasia and lymph node metastases [43], as confirmed by our results. Clinicopathological data are lacking to establish a strict comparison between DLL3 expression in the two available MTC cohorts [15, 43] and our cohort. As it happens in our cohort, in both series published, around half of the tumors had high expression of DLL3 (defined as ≥ 50% of positive tumor cells). In addition, the present study is the first one suggesting that DLL3 overexpression is associated with lower disease-free survival and overall survival. This contrasts with results from SCLC, where DLL3 expression has no impact on prognosis [14].

Our results may have relevance for MTC treatment. DLL3 is the target of several therapeutic agents that are currently under research in early development clinical trials in SCLC [44]. These agents include antibody–drug conjugates, T-cell engager molecules, and chimeric antigen receptor therapies. The first antibody–drug conjugate available was rovalpituzumab tenserine, a monoclonal anti-DLL3 antibody linked to a DNA intercalating agent (pyrrolobenzodiazepine) that exerts the cytotoxic action [15]. This conjugate was used in a phase I/II trial in 13 patients with MTC. In this trial, all MTC patients were recruited in stage IV; 6 (46%) expressing high DLL3 and 7 (54%) expressing low DLL3. However, trials with this drug were discontinued [44]. Among T-cell engager molecules, the most clinically advanced molecule is tarlatamab, a compound that bispecifically binds both DLL3 and CD3 leading to T-cell-mediated tumor lysis. It has already completed a phase 1 trial, and it is currently running a phase 2 study in SCLC [44, 45]. We hypothesize that patients with high-grade MTC, who exhibit a tendency towards high expression of DLL3 and an expression profile similar to SCLC-A, may benefit from inclusion in clinical trials using compounds that target DLL3.

Our results are limited by using a restricted gene panel designed to evaluate the tumor biology, microenvironment, and immune response. However, this approach has proven useful in demonstrating that morphological differences corresponding to tumor grade are associated with distinct expression profiles, which may be relevant for treatment strategies. In conclusion, we have identified a set of genes (*EGLN3*, *EXO1*, *UBE2T*, *UBE2C*, *FOXM1*, *CENPA*, *DLL3*, *CCNA2*, *SOX2*, *KIF23*, and *CDCA5*) differently expressed between high-grade and low-grade MTC. The main pathways altered were related to cell cycle progression. In addition, we have found that high-grade MTC upregulates *ASCL1*, *DLL3*, and *SOX2*, a gene signature akin to SCLC molecular subgroup A. Taking into consideration our results about the impact of DLL3 expression on prognosis, both desmoplasia and DLL3 overexpression should be explored as predictors of aggressive disease and poor outcomes.

## Supplementary Information

Below is the link to the electronic supplementary material.

Online Resource 1. Sequence of specific primers for qRT-PCR of cDNA samples and their location in the target genes (DOCX 15 KB)

Online Resource 2: Oncoplot of mutations identified in MTC tumors. Figure color code: high grade MTC (red) and low grade MTC (blue). TMB refers to the number of mutations identified by tumor (JPEG 110 KB)

Online Resource 3: Volcano plots showing genes differentially expressed according to (A) a Ki67 proliferation index ≥ 5% vs a proliferation index <5%; and (B) the presence of necrosis vs absence of necrosis (PPTX 81 KB)

Online Resource 4: (A) Correlation between DLL3 RNA expression and ASCL1 RNA expression quantified by qRT-PCR. (B) Correlation between SOX2 RNA expression and ASCL1 RNA expression quantified by qRT-PCR (JPG 549 KB)

## Data Availability

The datasets used and/or analyzed during the current study are available from the corresponding author on reasonable request.

## References

[1] Randle RW, Balentine CJ, Leverson GE, Havlena JA, Sippel RS, Schneider DF, Pitt SC (2017) Trends in the presentation, treatment, and survival of patients with medullary thyroid cancer over the past 30 years Surgery 161:137–146. 10.1016/j.surg.2016.04.053 10.1016/j.surg.2016.04.053 PMC5164945 27842913

[2] Le MK, Kawai M, Odate T, Vuong HG, Oishi N, Kondo T (2022) Metastatic Risk Stratification of 2526 Medullary Thyroid Carcinoma Patients: A Study Based on Surveillance, Epidemiology, and End Results Database Endocr Pathol 33:348-358. 10.1007/s12022-022-09724-2 35852678 10.1007/s12022-022-09724-2

[3] Xu B, Fuchs TL, Ahmadi S, Alghamdi M, Alzumaili B, Bani MA, Baudin E, Chou A, De Leo A, Fagin JA, Ganly I, Glover A, Hartl D, Kanaan C, Khneisser P, Najdawi F, Nigam A, Papachristos A, Repaci A, Spanheimer PM, Solaroli E, Untch BR, Barletta JA, Tallini G, Al Ghuzlan A, Gill AJ, Ghossein RA (2022) International Medullary Thyroid Carcinoma Grading System: A Validated Grading System for Medullary Thyroid Carcinoma J Clin Oncol 40:96-104. 10.1200/JCO.21.01329 34731032 10.1200/JCO.21.01329 PMC8683221

[4] Vissio E, Maletta F, Fissore J, Osella Abate S, Retta F, Brizzi MP, Piovesan A, Rossetto Giaccherino R, Volante M, Papotti M (2022) External Validation of Three Available Grading Systems for Medullary Thyroid Carcinoma in a Single Institution Cohort Endocr Pathol 33:359-370. 10.1007/s12022-022-09719-z 35583706 10.1007/s12022-022-09719-z

[5] Baloch ZW, Asa SL, Barletta JA, Ghossein RA, Juhlin CC, Jung CK, LiVolsi VA, Papotti MG, Sobrinho-Simoes M, Tallini G, Mete O (2022) Overview of the 2022 WHO Classification of Thyroid Neoplasms Endocr Pathol 33:27-63. 10.1007/s12022-022-09707-3 35288841 10.1007/s12022-022-09707-3

[6] Ciampi R, Romei C, Ramone T, Prete A, Tacito A, Cappagli V, Bottici V, Viola D, Torregrossa L, Ugolini C, Basolo F, Elisei R (2019) Genetic Landscape of Somatic Mutations in a Large Cohort of Sporadic Medullary Thyroid Carcinomas Studied by Next-Generation Targeted Sequencing iScience 20:324-336. 10.1016/j.isci.2019.09.030 10.1016/j.isci.2019.09.030 PMC6817656 31605946

[7] Xu B, Viswanathan K, Ahadi MS, Ahmadi S, Alzumaili B, Bani MA, Baudin E, Behrman DB, Capelletti M, Chau NG, Chiarucci F, Chou A, Clifton-Bligh R, Coluccelli S, de Biase D, De Leo A, Dogan S, Fagin JA, Fuchs TL, Glover AR, Hadoux J, Lacroix L, Lamartina L, Lubin DJ, Luxford C, Magliocca K, Maloberti T, Mohanty AS, Najdawi F, Nigam A, Papachristos AJ, Repaci A, Robinson B, Scoazec JY, Shi Q, Sidhu S, Solaroli E, Sywak M, Tuttle RM, Untch B, Barletta JA, Al Ghuzlan A, Gill AJ, Ghossein R, Tallini G, Ganly I (2024) Association of the Genomic Profile of Medullary Thyroid Carcinoma with Tumor Characteristics and Clinical Outcomes in an International Multicenter Study Thyroid 34:167-176. 10.1089/thy.2023.0279 37842841 10.1089/thy.2023.0279 PMC10884546

[8] Censi S, Galuppini F, Clausi C, Battheu F, Manso J, Piva I, Corvaglia S, Pedron MC, Mondin A, Iacobone M, Torresan F, Merante Boschin I, Bertazza L, Barollo S, Pennelli G, Mian C (2024) Tumor Grade and Molecular Characteristics Associated with Survival in Sporadic Medullary Thyroid Carcinoma Thyroid 34:177-185. 10.1089/thy.2023.0482 38047536 10.1089/thy.2023.0482

[9] Maliszewska A, Leandro-Garcia LJ, Castelblanco E, Macia A, de Cubas A, Gomez-Lopez G, Inglada-Perez L, Alvarez-Escola C, De la Vega L, Leton R, Gomez-Grana A, Landa I, Cascon A, Rodriguez-Antona C, Borrego S, Zane M, Schiavi F, Merante-Boschin I, Pelizzo MR, Pisano DG, Opocher G, Matias-Guiu X, Encinas M, Robledo M (2013) Differential gene expression of medullary thyroid carcinoma reveals specific markers associated with genetic conditions Am J Pathol 182:350-362. 10.1016/j.ajpath.2012.10.025 23201134 10.1016/j.ajpath.2012.10.025

[10] Mancikova V, Montero-Conde C, Perales-Paton J, Fernandez A, Santacana M, Jodkowska K, Inglada-Perez L, Castelblanco E, Borrego S, Encinas M, Matias-Guiu X, Fraga M, Robledo M (2017) Multilayer OMIC Data in Medullary Thyroid Carcinoma Identifies the STAT3 Pathway as a Potential Therapeutic Target in RET(M918T) Tumors Clin Cancer Res 23:1334–1345. 10.1158/1078-0432.CCR-16-0947 10.1158/1078-0432.CCR-16-0947 27620278

[11] Qu N, Shi X, Zhao JJ, Guan H, Zhang TT, Wen SS, Liao T, Hu JQ, Liu WY, Wang YL, Huang S, Shi RL, Wang Y, Ji QH (2020) Genomic and Transcriptomic Characterization of Sporadic Medullary Thyroid Carcinoma Thyroid 30:1025-1036. 10.1089/thy.2019.0531 32031055 10.1089/thy.2019.0531

[12] Koperek O, Scheuba C, Cherenko M, Neuhold N, De Micco C, Schmid KW, Niederle B, Kaserer K (2008) Desmoplasia in medullary thyroid carcinoma: a reliable indicator of metastatic potential Histopathology 52:623-630. 10.1111/j.1365-2559.2008.03002.x 18370959 10.1111/j.1365-2559.2008.03002.x

[13] NanoString. nCounter® Tumor Signaling 360 panel. . https://nanostring.com/products/ncounter-assays-panels/oncology/tumor-signaling-360/. Accessed February 9, 2024 2024

[14] Rojo F, Corassa M, Mavroudis D, Oz AB, Biesma B, Brcic L, Pauwels P, Sailer V, Gosney J, Miljkovic D, Hader C, Wu M, Almarez T, Penault-Llorca F (2020) International real-world study of DLL3 expression in patients with small cell lung cancer Lung Cancer 147:237–243. 10.1016/j.lungcan.2020.07.026 10.1016/j.lungcan.2020.07.026 32745892

[15] Mansfield AS, Hong DS, Hann CL, Farago AF, Beltran H, Waqar SN, Hendifar AE, Anthony LB, Taylor MH, Bryce AH, Tagawa ST, Lewis K, Niu J, Chung CH, Cleary JM, Rossi M, Ludwig C, Valenzuela R, Luo Y, Aggarwal R (2021) A phase I/II study of rovalpituzumab tesirine in delta-like 3-expressing advanced solid tumors NPJ Precis Oncol 5:74. 10.1038/s41698-021-00214-y 10.1038/s41698-021-00214-y PMC8342450 34354225

[16] Owen DH, Giffin MJ, Bailis JM, Smit MD, Carbone DP, He K (2019) DLL3: an emerging target in small cell lung cancer J Hematol Oncol 12:61. 10.1186/s13045-019-0745-2 10.1186/s13045-019-0745-2 PMC6582566 31215500

[17] Gomaa W, Marouf A, Alamoudi A, Al-Maghrabi J (2020) SOX2 Is a Potential Novel Marker of Undifferentiated Thyroid Carcinomas Cureus 12:e12102. 10.7759/cureus.12102 10.7759/cureus.12102 PMC7805510 33489519

[18] Henke RM, Meredith DM, Borromeo MD, Savage TK, Johnson JE (2009) Ascl1 and Neurog2 form novel complexes and regulate Delta-like3 (Dll3) expression in the neural tube Dev Biol 328:529–540. 10.1016/j.ydbio.2009.01.007 10.1016/j.ydbio.2009.01.007 PMC2698949 19389376

[19] Rudin CM, Poirier JT, Byers LA, Dive C, Dowlati A, George J, Heymach JV, Johnson JE, Lehman JM, MacPherson D, Massion PP, Minna JD, Oliver TG, Quaranta V, Sage J, Thomas RK, Vakoc CR, Gazdar AF (2019) Molecular subtypes of small cell lung cancer: a synthesis of human and mouse model data Nat Rev Cancer 19:289-297. 10.1038/s41568-019-0133-9 30926931 10.1038/s41568-019-0133-9 PMC6538259

[20] Ireland AS, Micinski AM, Kastner DW, Guo B, Wait SJ, Spainhower KB, Conley CC, Chen OS, Guthrie MR, Soltero D, Qiao Y, Huang X, Tarapcsak S, Devarakonda S, Chalishazar MD, Gertz J, Moser JC, Marth G, Puri S, Witt BL, Spike BT, Oliver TG (2020) MYC Drives Temporal Evolution of Small Cell Lung Cancer Subtypes by Reprogramming Neuroendocrine Fate Cancer Cell 38:60-78 e12. 10.1016/j.ccell.2020.05.001 10.1016/j.ccell.2020.05.001 PMC7393942 32473656

[21] Strocchi S, Reggiani F, Gobbi G, Ciarrocchi A, Sancisi V (2022) The multifaceted role of EGLN family prolyl hydroxylases in cancer: going beyond HIF regulation Oncogene 41:3665-3679. 10.1038/s41388-022-02378-8 35705735 10.1038/s41388-022-02378-8

[22] Li S, Rodriguez J, Li W, Bullova P, Fell SM, Surova O, Westerlund I, Topcic D, Bergsland M, Stenman A, Muhr J, Nister M, Holmberg J, Juhlin CC, Larsson C, von Kriegsheim A, Kaelin WG, Jr., Schlisio S (2019) EglN3 hydroxylase stabilizes BIM-EL linking VHL type 2C mutations to pheochromocytoma pathogenesis and chemotherapy resistance Proc Natl Acad Sci U S A 116:16997–17006. 10.1073/pnas.1900748116 10.1073/pnas.1900748116 PMC6708352 31375625

[23] Rodriguez J, Herrero A, Li S, Rauch N, Quintanilla A, Wynne K, Krstic A, Acosta JC, Taylor C, Schlisio S, von Kriegsheim A (2018) PHD3 Regulates p53 Protein Stability by Hydroxylating Proline 359 Cell Rep 24:1316–1329. 10.1016/j.celrep.2018.06.108 10.1016/j.celrep.2018.06.108 PMC6088137 30067985

[24] Tanaka T, Torigoe T, Hirohashi Y, Sato E, Honma I, Kitamura H, Masumori N, Tsukamoto T, Sato N (2014) Hypoxia-inducible factor (HIF)-independent expression mechanism and novel function of HIF prolyl hydroxylase-3 in renal cell carcinoma J Cancer Res Clin Oncol 140:503–513. 10.1007/s00432-014-1593-7 10.1007/s00432-014-1593-7 PMC11823785 24477694

[25] Andersen S, Donnem T, Stenvold H, Al-Saad S, Al-Shibli K, Busund LT, Bremnes RM (2011) Overexpression of the HIF hydroxylases PHD1, PHD2, PHD3 and FIH are individually and collectively unfavorable prognosticators for NSCLC survival PLoS One 6:e23847. 10.1371/journal.pone.0023847 10.1371/journal.pone.0023847 PMC3161788 21887331

[26] Dopeso H, Jiao HK, Cuesta AM, Henze AT, Jurida L, Kracht M, Acker-Palmer A, Garvalov BK, Acker T (2018) PHD3 Controls Lung Cancer Metastasis and Resistance to EGFR Inhibitors through TGFalpha Cancer Res 78:1805–1819. 10.1158/0008-5472.CAN-17-1346 10.1158/0008-5472.CAN-17-1346 29339541

[27] Ivan M, Kaelin WG, Jr. (2017) The EGLN-HIF O(2)-Sensing System: Multiple Inputs and Feedbacks Mol Cell 66:772–779. 10.1016/j.molcel.2017.06.002 10.1016/j.molcel.2017.06.002 PMC5613951 28622522

[28] Lodewijk L, van Diest P, van der Groep P, Ter Hoeve N, Schepers A, Morreau J, Bonenkamp J, van Engen-van Grunsven A, Kruijff S, van Hemel B, Links T, Nieveen van Dijkum E, van Eeden S, Valk G, Borel Rinkes I, Vriens M (2017) Expression of HIF-1alpha in medullary thyroid cancer identifies a subgroup with poor prognosis Oncotarget 8:28650–28659. 10.18632/oncotarget.15622 10.18632/oncotarget.15622 PMC5438680 28404916

[29] Jiang A, Zhou Y, Gong W, Pan X, Gan X, Wu Z, Liu B, Qu L, Wang L (2022) CCNA2 as an Immunological Biomarker Encompassing Tumor Microenvironment and Therapeutic Response in Multiple Cancer Types Oxid Med Cell Longev 2022:5910575. 10.1155/2022/5910575 10.1155/2022/5910575 PMC8989596 35401923

[30] Barger CJ, Branick C, Chee L, Karpf AR (2019) Pan-Cancer Analyses Reveal Genomic Features of FOXM1 Overexpression in Cancer Cancers (Basel) 11. 10.3390/cancers11020251 10.3390/cancers11020251 PMC6406812 30795624

[31] Hao Z, Zhang H, Cowell J (2012) Ubiquitin-conjugating enzyme UBE2C: molecular biology, role in tumorigenesis, and potential as a biomarker Tumour Biol 33:723–730. 10.1007/s13277-011-0291-1 10.1007/s13277-011-0291-1 22170434

[32] Xiang C, Yan HC (2022) Ubiquitin conjugating enzyme E2 C (UBE2C) may play a dual role involved in the progression of thyroid carcinoma Cell Death Discov 8:130. 10.1038/s41420-022-00935-4 10.1038/s41420-022-00935-4 PMC8948250 35332135

[33] Takahashi K, Yamanaka S (2006) Induction of pluripotent stem cells from mouse embryonic and adult fibroblast cultures by defined factors Cell 126:663-676. 10.1016/j.cell.2006.07.024 16904174 10.1016/j.cell.2006.07.024

[34] Novak D, Huser L, Elton JJ, Umansky V, Altevogt P, Utikal J (2020) SOX2 in development and cancer biology Semin Cancer Biol 67:74–82. 10.1016/j.semcancer.2019.08.007 10.1016/j.semcancer.2019.08.007 31412296

[35] Wang S, Liu X, Chen Y, Zhan X, Wu T, Chen B, Sun G, Yan S, Xu L (2020) The role of SOX2 overexpression in prognosis of patients with solid tumors: A meta-analysis and system review Medicine (Baltimore) 99:e19604. 10.1097/MD.0000000000019604 10.1097/MD.0000000000019604 PMC7220337 32221082

[36] Gauchotte G, Philippe C, Lacomme S, Leotard B, Wissler MP, Allou L, Toussaint B, Klein M, Vignaud JM, Bressenot A (2011) BRAF, p53 and SOX2 in anaplastic thyroid carcinoma: evidence for multistep carcinogenesis Pathology 43:447–452. 10.1097/PAT.0b013e3283486178 10.1097/PAT.0b013e3283486178 21716161

[37] Marshall OJ, Marshall AT, Choo KH (2008) Three-dimensional localization of CENP-A suggests a complex higher order structure of centromeric chromatin J Cell Biol 183:1193-1202. 10.1083/jcb.200804078 19114591 10.1083/jcb.200804078 PMC2606971

[38] Roulland Y, Ouararhni K, Naidenov M, Ramos L, Shuaib M, Syed SH, Lone IN, Boopathi R, Fontaine E, Papai G, Tachiwana H, Gautier T, Skoufias D, Padmanabhan K, Bednar J, Kurumizaka H, Schultz P, Angelov D, Hamiche A, Dimitrov S (2016) The Flexible Ends of CENP-A Nucleosome Are Required for Mitotic Fidelity Mol Cell 63:674-685. 10.1016/j.molcel.2016.06.023 27499292 10.1016/j.molcel.2016.06.023

[39] Liverani C, Bongiovanni A, Mercatali L, Pieri F, Spadazzi C, Miserocchi G, Di Menna G, Foca F, Ravaioli S, De Vita A, Cocchi C, Rossi G, Recine F, Ibrahim T (2021) Diagnostic and Predictive Role of DLL3 Expression in Gastroenteropancreatic Neuroendocrine Neoplasms Endocr Pathol 32:309–317. 10.1007/s12022-020-09657-8 10.1007/s12022-020-09657-8 33409812

[40] Borromeo MD, Savage TK, Kollipara RK, He M, Augustyn A, Osborne JK, Girard L, Minna JD, Gazdar AF, Cobb MH, Johnson JE (2016) ASCL1 and NEUROD1 Reveal Heterogeneity in Pulmonary Neuroendocrine Tumors and Regulate Distinct Genetic Programs Cell Rep 16:1259–1272. 10.1016/j.celrep.2016.06.081 10.1016/j.celrep.2016.06.081 PMC4972690 27452466

[41] Niederle MB, Riss P, Selberherr A, Koperek O, Kaserer K, Niederle B, Scheuba C (2021) Omission of lateral lymph node dissection in medullary thyroid cancer without a desmoplastic stromal reaction Br J Surg 108:174-181. 10.1093/bjs/znaa047 33704404 10.1093/bjs/znaa047

[42] Gomez-Ramirez J, Luengo P, Mercader E, Quintana A, Munoz de Nova JL, Febrero B, Ruz-Caracuel I, Rodriguez JM (2023) Desmoplastic reaction in medullary thyroid carcinoma predicts presence of lymph node metastasis Br J Surg 110:1011-1012. 10.1093/bjs/znad172 37343053 10.1093/bjs/znad172

[43] Ingenwerth M, Brandenburg T, Fuhrer-Sakel D, Goetz M, Weber F, Dralle H, Schildhaus HU, Schmid KW, Theurer S (2021) DLL3 (delta-like protein 3) expression correlates with stromal desmoplasia and lymph node metastases in medullary thyroid carcinomas Endocr Connect 10:283–289. 10.1530/EC-20-0611 10.1530/EC-20-0611 PMC8052580 33617464

[44] Rudin CM, Reck M, Johnson ML, Blackhall F, Hann CL, Yang JC, Bailis JM, Bebb G, Goldrick A, Umejiego J, Paz-Ares L (2023) Emerging therapies targeting the delta-like ligand 3 (DLL3) in small cell lung cancer J Hematol Oncol 16:66. 10.1186/s13045-023-01464-y 10.1186/s13045-023-01464-y PMC10290806 37355629

[45] Paz-Ares L, Champiat S, Lai WV, Izumi H, Govindan R, Boyer M, Hummel HD, Borghaei H, Johnson ML, Steeghs N, Blackhall F, Dowlati A, Reguart N, Yoshida T, He K, Gadgeel SM, Felip E, Zhang Y, Pati A, Minocha M, Mukherjee S, Goldrick A, Nagorsen D, Hashemi Sadraei N, Owonikoko TK (2023) Tarlatamab, a First-in-Class DLL3-Targeted Bispecific T-Cell Engager, in Recurrent Small-Cell Lung Cancer: An Open-Label, Phase I Study J Clin Oncol 41:2893-2903. 10.1200/JCO.22.02823 36689692 10.1200/JCO.22.02823 PMC10414718

