# Supplementary material for: Transcriptomic Differences in Medullary Thyroid Carcinoma According to Grade
Source: Endocr Pathol. 2024 Jul 3;35(3):207–18. doi: 10.1007/s12022-024-09817-0 (PMC11387449; doi:10.1007/s12022-024-09817-0)
Supplement: Supplementary file 1 — Online Resource 1. Sequence of specific primers for qRT-PCR of cDNA samples and their location in the target genes (DOCX 15 KB) [file 12022_2024_9817_MOESM1_ESM.docx]

| **Supplementary table 1.**  **Sequence of specific primers for qRT-PCR of cDNA samples and their location in the target genes** | | | | |
| --- | --- | --- | --- | --- |
| **Gene name** | **Forward primer location** | **Forward primer sequence** | **Reverse primer location** | **Reverse primer sequence** |
| cDNA *UBE2C* | *UBE2C* exon 1 | 5´ CGTAAAGGAGCTGAGCCGAG 3´ | *UBE2C* exon 3 | 5´ TCAGGGAAGGCAGAAATCCC 3´ |
| cDNA *FOXM1* | *FOXM1* exon 2 | 5´ TAGACCACCTGGAGCCCTTT 3´ | *FOXM1* exon 3 | 5´ GGAGCCCAGTCCATCAGAAC 3´ |
| cDNA *CCNA2* | *CCNA2* exon 2 | 5´ ACCTGGACCCAGAAAACCATT 3´ | *CCNA2* exon 3 | 5´ CACTCACTGGCTTTTCATCTTCT 3´ |
| cDNA *CDCA5* | *CDCA5* exon 3-4 | 5´ GAGGTCCCAGCTGTCCAATC 3´ | *CDCA5* exon 5 | 5´ GGGACGCTGTGTGTCTTGAA 3´ |
| cDNA *EGLN3* | *EGLN3* exon 1 | 5´ ATCGACAGGCTGGTCCTCTA 3´ | *EGLN3* exon 2 | 5´ GTCCACGTGGCGAACATAAC 3´ |
| cDNA *DLL3* | *DLL3*  exon 4 | 5´ CGCTCGAGGACGAATGTGAG 3´ | *DLL3*  exon 5 | 5´ CCCTCTAGGCATCGGCATTC 3´ |
| cDNA *SOX2* | *SOX2* exon 1 | 5´ CAGCTCGCAGACCTACATGA 3´ | *SOX2* exon 1 | 5´ CTCGGACTTGACCACCGAAC 3´ |
| cDNA *ASCL1* | *ASCL1* exon 1 | 5´ GAACTGATGCGCTGCAAACG 3´ | *ASCL1* exon 1 | 5´ TTGACCAACTTGACGCGGTT 3´ |
| cDNA *GAPDH* (ref. gene) | *GAPDH* exon 3 | 5´ GGTCACCAGGGCTGCTTTTA 3´ | *GAPDH* exon 4-5 | 5´ GGGATCTCGCTCCTGGAAGA 3´ |
