# Supplementary figures and images for: Transcriptomic Differences in Medullary Thyroid Carcinoma According to Grade
Source: Endocr Pathol. 2024 Jul 3;35(3):207–18. doi: 10.1007/s12022-024-09817-0 (PMC11387449; doi:10.1007/s12022-024-09817-0)

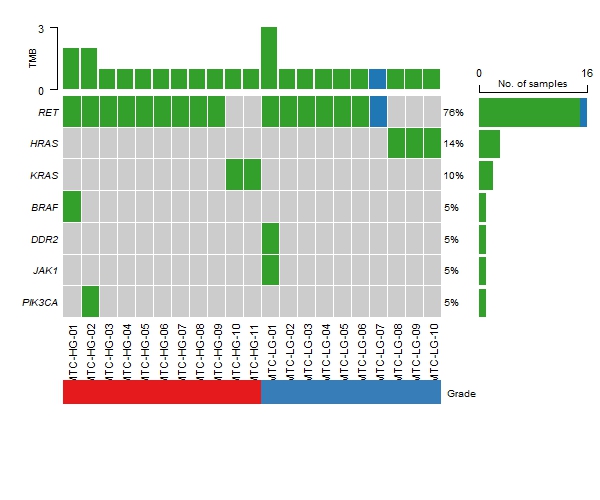

Supplement: Supplementary file 2 — Online Resource 2: Oncoplot of mutations identified in MTC tumors. Figure color code: high grade MTC (red) and low grade MTC (blue). TMB refers to the number of mutations identified by tumor (JPEG 110 KB) [file 12022_2024_9817_MOESM2_ESM.jpeg]

## Slide 1
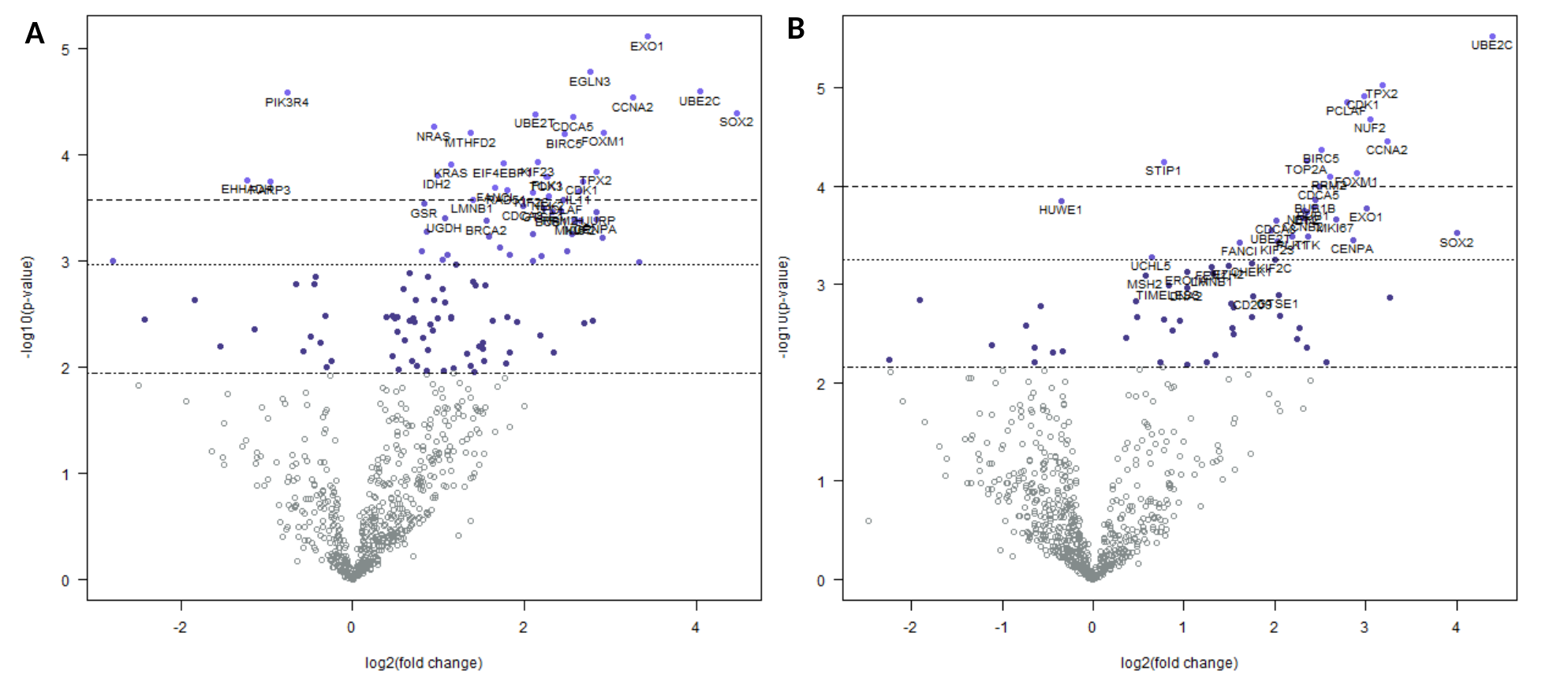

B
A

Supplement: Supplementary file 3 — Online Resource 3: Volcano plots showing genes differentially expressed according to (A) a Ki67 proliferation index ≥ 5% vs a proliferation index <5%; and (B) the presence of necrosis vs absence of necrosis (PPTX 81 KB) [file 12022_2024_9817_MOESM3_ESM.pptx]

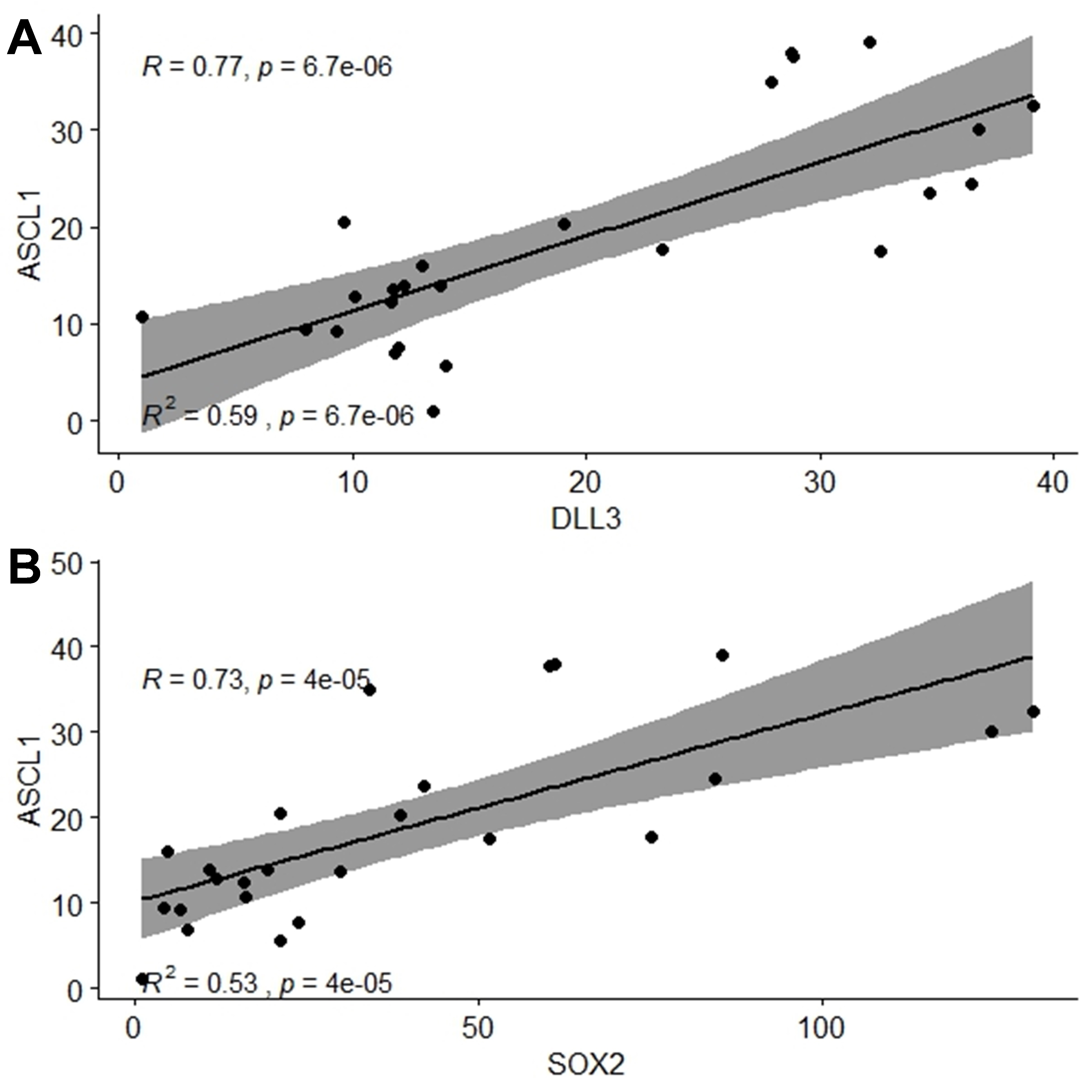

Supplement: Supplementary file 4 — Online Resource 4: (A) Correlation between DLL3 RNA expression and ASCL1 RNA expression quantified by qRT-PCR. (B) Correlation between SOX2 RNA expression and ASCL1 RNA expression quantified by qRT-PCR (JPG 549 KB) [file 12022_2024_9817_MOESM4_ESM.jpg]
